# The effect of gender and menstrual cycle phase on patıents undergoıng ımpacted thırd molar surgery: a cross-sectıonal study

**DOI:** 10.4317/medoral.26443

**Published:** 2024-06-22

**Authors:** Nida Geçkil

**Affiliations:** 1 Niğde Ömer Halisdemir University, Faculty of Dentistry, Niğde, Turkey

## Abstract

**Background:**

Achieving the best outcomes in surgical procedures requires optimizing all patient-related psychological and physiological factors. This study was carried out to evaluate the preoperative anxiety and fear levels, and postoperative symptoms in patients undergoing impacted third molar surgery, and to compare the relevant psychological and physical findings between genders and between women in different menstrual cycle phases.

**Material and Methods:**

The population of this prospective and clinical study consisted of patients who applied to faculty of dentistry for the extraction of impacted third molars. The menstrual cycles of the female patients included in the study ranged from 26 to 32 days. The female patients included in the study were divided into three groups according to the first day of the menstrual cycle and bleeding status. All patients were administered Spielberger State-Trait Anxiety Inventory Short Version (STAI-S), Dental Fear Survey (DFS), Modified Dental Anxiety Scale (MDAS) preoperatively, and postoperative satisfaction and complication questionnaires.

**Results:**

The mean age of the 128 patients included in the study was 27.04±4.62 years. Of these patients, 79 (61.7%) were female and 49 (38.3%) were male. Female patients had significantly higher STAI-S, MDAS and DFS scores than male patients (94 vs. 53; 16 vs. 9; 58 vs. 27; *p*<0.001, respectively). In parallel, female patients had significantly higher complication rates, thus significantly lower satisfaction levels than male patients (116 vs. 51; 40 vs. 13; *p*<0.001, respectively). STAI-S, MDAS and DFS scores were high in women during the secretory phase (*p*<0.001). In the secretory phase, complications were high and satisfaction was low (*p*<0.001).

**Conclusions:**

The findings of the study reveal that women have a harder time getting through the operation process and that timing is important in reducing preoperative anxiety and fear levels and increasing postoperative satisfaction levels and complication rates.

** Key words:**Oral surgery, sex hormones, inflammation, pain.

## Introduction

Dental procedures are one of the procedures that trigger anxiety and fear the most among interventional approaches. Among dental procedures, it is known that dental surgeries are the scene of the highest levels of anxiety and fear (1). The anxiety and fear experienced before dental procedures is generally referred to as dental anxiety. The prevalence of dental anxiety in the general population varies between 4% and 23.4% (2). Studies on strategies for coping with dental anxiety have shown that establishing straightforward communication with patients and providing them with information about dental procedures reduces dental anxiety (3,4).

In addition to environmental factors, hormones also directly affect patients’ anxiety levels. Momentary emotional states and physiological and psychological responses to physical stimuli are induced by the hormones (5). While hormone levels are more sTable in men, the dominant hormones in women change periodically within an average of 28 days (6). The menstrual cycle phase of regularly menstruating women can be accurately determined based on an analysis of the menstrual cycles in three phases according to the endometrium. Since the functional layer of the endometrium changes under the influence of hormones released from the ovaries, the cycle phase can be estimated according to the onset of bleeding (7). Endometrial phases begin with the menstrual phase on the first day of menstruation. The menstrual phase, which features the shedding of the functional layer of the endometrium with bleeding, lasts 4-5 days. It is characterised by low levels of estrogen and progesterone. Inguinal pain, feeling of fullness in the lower abdomen and hypoglycaemia may be observed (7). The next phase is the proliferative phase. It is characterised by the increase in the level of estrogen hormone secreted from the follicles developing in the ovaries. During this phase, proliferation is observed in stromal cells, the blood supply of the endometrium is increased, and the uterine wall gets thicker. The proliferative phase featuring the dominance of estrogen continues until the 14th day of the menstrual cycle after bleeding ends. After the 14th day of the menstrual cycle, the secretory phase begins with ovulation. During this phase, progesterone level and mucus secretion increase and the endometrium wall gets thicker. The secretory phase continues until the day when bleeding starts again. Depressive mood, tension and restlessness often accompany this phase towards its last days in the premenstrual period (7).

These hormonal changes affect the anxiety and pain perception levels, as well as the inflammatory response of the body in women (5). Hormonal differences between genders and between menstrual cycle phases in women warrant investigating dental anxiety in these contexts. However, a thorough literature review did not reveal any study on the relationship between hormonal status, dental anxiety and postoperative inflammatory response.

In light of this information, this study was carried out to evaluate the preoperative anxiety and fear levels, and postoperative symptoms in patients undergoing impacted mandibular third molar tooth extraction surgery, which is considered to trigger anxiety the most among dental procedures, between genders and women in different menstrual cycle phases. To this end, patients’ hormonal status-related psychological, emotional and physical reactions to dental surgery were compared using subjective and universal scales for the first time in the literature.

## Material and Methods

- Study Design

The study protocol was approved by Niğde Ömer Halisdemir University Clinical Research Ethics Committee (Approval No: 2023/37, Approval Date: July 13th, 2023). The study was carried out in accordance with the ethical principles set forth in the Declaration of Helsinki. Patients were informed about the risks and requirements of the surgical procedure prior to the conduct of the study. To this end, written informed consent form was obtained from each patient participated in the study.

All the preoperative and postoperative work and data collection was done by a single experienced surgeon (NG). Since it was a multifactorial study, the STROBE checklist was prepared and the same procedure sequence was followed in all patients.

- Population and Sample

The study population consisted of patients aged between 20 and 35 years, who applied to Niğde Ömer Halisdemir University, Faculty of Dentistry, Department of Oral, Dental and Maxillofacial Surgery between July and September 2023 for the extraction of class II and position B impacted third molars. Patients with systemic diseases, psychological disorders or cognitive difficulties, obesity, previous surgical operations, menstrual irregularities, patients using medications including oral contraceptives, and patients who might be pregnant were excluded from the study. Female patients whose menstrual cycle did not last between 26 and 32 days in the last three months were also excluded from the study. Additionally, since it is known that the duration and difficulty of the surgical procedure affects the inflammatory response after the procedure, only patients whose impacted third molar surgery was predicted to be of similar difficulty were included in the study. Accordingly, only patients with class II impacted third molars in the B position were included in the study. The class and position of the impacted third molars were determined based on the Pell and Gregory system (8) and Archer classification (9), respectively.

- Preoperative Procedures

Patients who were determined to be suiTable for the study were given an appointment and asked to be at the clinic at least half an hour before the surgery. Patients’ demographic information was queried. In addition, female patients were queried about their last menstrual cycle onset date and and the phase of their menstrual cycle, based on whether there was bleeding. All patients were administered Spielberger State-Trait Anxiety Inventory Short Version (STAI-S), Dental Fear Survey (DFS), and Modified Dental Anxiety Scale (MDAS) preoperatively. Before the surgical operation, the patients were seated on a chair and covered with a sterile drape.

Spielberger State-Trait Anxiety Inventory Short Version (STAI-S):

The STAI-S, developed by Spielberger (10) is one of the most frequently used self-report assessment tools in anxiety research. The inventory, which is not specific to dental anxiety, has two subscales, namely state and trait anxiety subscales, each consisting of 20 items. The state anxiety subscale assesses patient’s current anxiety level, whereas trait anxiety subscale assesses patient’s underlying (ongoing/personality) anxiety level. The total score that can be obtained from the 4-point Likert-type inventory ranges between 20 and 80. Scores between 20 and 37 indicae no or low anxiety, 38 and 44 indicate moderate anxiety, and 45 and 80 indicate high anxiety (4,11).

Dental Fear Scale (DFS):

The DFS, developed by Kleinknecht *et al*. (12), is used to assess a patient’s dental fear in different dimensions. The 5-point Likert-type scale consists of 20 items examining the levels of fear in terms of dentist avoidance, somatic symptoms of fear, and fear of various applications in dentistry practice. Total possible scores on DFS range from 20 to 100. Higher scores indicate higher levels of dental fear (12,13).

Modified Dental Anxiety Scale (MDAS):

MDAS was developed by Humphris *et al*. (14) by adding an item on anxiety about oral injections to the 4-item Dental Anxiety Scale developed by N. L. Corah in 1969. The total score that can be obtained from the 5-item and 5-point Likert-type scale ranges between 5 and 25. Scores between 5 and 11 indicae no or low anxiety, 12 and 18 indicate moderate anxiety, and 19 and 25 indicate high anxiety (15).

- Surgical Procedure

In all patients, inferior alveolar nerve block and buccal anesthesia were achieved by injecting 4% articaine with 1:100,000 epinephrine in a single-use plastic disposable 2mL syringe. Within the scope of the surgical procedure, firstly, a distal releasing Winter type incision was made in all patients. The flap was lifted and the buccal bone was exposed. Osteotomy were performed with standard tungsten carbide burs under abundant irrigation. After tooth extraction, the socket was curetised and washed. Once the bleeding was controlled, the flap was closed with 3.0 silk suture. The whole procedure took 30 to 40 minutes.

- Postoperative Procedures

Postoperatively, all patients were prescribed paracetamol 500 mg twice daily, amoxicillin 1000 mg twice daily and chlorhexidine gluconate-benzidamine hydrochloride antiseptic mouthwash to be used three times daily. Oral hygiene training was given and post-operative care recommendations were presented orally and in writing.

All patients were given satisfaction (Table 1) and complication (Table 2) assessment questionnaires to answer after the procedure and to return one week later for suture removal and clinical follow-up.

Postoperative Satisfaction Questionnaire (PSQ):

The patients were given a 7-point Likert-type PSQ after the surgery to fill out and return on the 7th postoperative day (16). The first eight questions in the questionnaire assess the severity of inflammation, the following eight questions assess any difficulty experienced during daily activities, and the last four questions assess patient satisfaction. As the score increases on this scale, the satisfaction level decreases.

Postoperative Complication Questionnaire (PCQ):

The patients were given a 7-point Likert-type PCQ after the surgery to fill out and return on the 7th postoperative day (17). The eight-item questionnaire includes questions that assess trismus, pain, swelling, bleeding and nerve damage. In the evaluation, it is taken into account that the degree of complication increases as the scale score increases.

- Statistical Analysis

The compatibility of the variables with normal distribution was examined by Shapiro-Wilk test. Continuous variables were expressed as median [minimum-maximum] and categorical variables as n (%). According to the normality test results, Mann-Whitney U test was used for comparisons between two groups. Kruskal-Wallis H test was used for comparisons between three or more groups. Chi-Square test was used to compare categorical variables. Spearman correlation coefficient was used to analyse the relationship between the scales. According to the findings obtained from similar study, when the effect size was 0.23 (18), the significance level was 0.05 and the power of the study was 0.80, the minimum required sample size was determined as 119 people. The required sample size was calculated using G*Power 3.1.9.4 programme. Statistical analysis was performed using SPSS (IBM Corp. Released 2017. Version 25.0) and *p*<0.05 was considered statistically significant.

In the reliability analysis of the Satisfaction and complication questionnaires, Cronbach's alpha internal consistency coefficients were calculated as 0.981 and 0.964, respectively. According to the results of exploratory factor analysis to determine the construct validity of both scales, Kaiser-Meyer-Olkin coefficients were 0.946 and 0.894, respectively. As a result of Bartlett's test, chi-square values were calculated as 3700.88 (*p*<0.001) and 1212.84 (*p*<0.001), respectively. It was determined that the sample size was sufficient for factor analysis. The ratios of explaining the total variance in both scale items were obtained as 73.82% and 79.94% in one dimension, respectively. According to this result, scale items can be scored in a single point.

## Results

The mean age of the patients participating in the study was 27.04±4.62 years. Of the 128 patients included in the study, 79 (61.7%) were female and 49 (38.3%) were male. The mean menstural cycle of female patients was 29.65±1.58 days. Of these 79 female patients, 23 (29.1%) were in the menstrual phase, 26 (32.9%) in the proliferative phase, and 30 (38%) in the secretory phase.

The median STAI total score was significantly higher in female patients than in male patients (94 vs. 63; *p*<0.001). The median STAI state and trait anxiety subscale scores were also significantly higher in female patients than in male patients (49 vs. 33; and 48 vs. 31; respectively; *p*<0.001 for both cases). Similarly, the median DFS total score was significantly higher in female patients than in male patients (58 vs. 27; *p*<0.001). In parallel, the median MDAS total score was significantly higher in female patients than in male patients (16 vs. 9; *p*<0.001). Of the patients with a total MDAS score of 19 and above, 3.3% were male. Female patients with a total MDAS score of 19 and above constituted 96.7%. In sum, preoperative anxiety and fear levels of female patients were higher than male patients (Table 3).

The median PSQ and PCQ total scores were significantly higher in female patients than in male patients (116 vs. 51; and 40 vs. 13; respectively; *p*<0.001 for both cases). Accordingly, it was determined that female patients had significantly lower postoperative satisfaction levels and significantly higher complication rates than male patients (Table 3).

There were significant differences between female patients in different menstrual cycle phases in total STAI scores as well as in STAI state and trait anxiety subscale scores (*p*<0.001 for all cases). The median total STAI score of female patients in menstrual, proliferative, and secretory phases was 100.5, 87, and 147, respectively. Similarly, there were significant differences between female patients in different menstrual cycle phases in total DFS scores (*p*<0.001 for all cases). Accordingly, the median total DFS score of female patients in the secretory phase was significantly higher than those in menstrual and proliferative phases. In parallel, there were significant differences between female patients in different menstrual cycle phases in total MDAS scores (*p*<0.001 for all cases). The median total MDAS score of female patients in menstrual, proliferative, and secretory phases was 17, 12, and 20, respectively. In sum, women in the secretory phase had the highest level of anxiety and women in the proliferative phase had the lowest level of anxiety (Table 4).

The median total PSQ and PCQ scores have also differed significantly between female patients in different menstrual cycle phases (*p*<0.001 for all cases). The median total PSQ score of female patients in menstrual, proliferative, and secretory phases was 51, 79, and 35, respectively; whereas the median total PCQ score of female patients in menstrual, proliferative, and secretory phases was 42, 22, and 49, respectively. The scores clearly showed that postoperative satisfaction levels were significantly lower and complications were significantly higher in female patients in the secretory phase (Table 4).

## Discussion

Women start menstruating with the onset of puberty (18). Periodic changes in hormones due to the menstrual cycle, which lasts approximately 28 days, lead to psychological, physiological and emotional changes in women. Hormonal differences between the genders and between women in different menstrual cycle phases have been extensively studied (19). As part of these studies, it was determined that women's hormonal states during dental treatments were associated with their pain perception and stress levels (20). However, to date, there is no study investigating the differences in pain perception and stress levels due to oral surgery between genders and between women in different phases of the menstrual cycle.

The criteria that must be met for a surgical procedure to be considered successful include accurate management of patient psychology, use of appropriate surgical techniques, and ensuring patient’s comfort and satisfaction. Accordingly, a successful surgical procedure starts with controlling the patient's preoperative anxiety (20). In a systemic meta-analysis study conducted in 2021, Silveira *et al*. (21) reported that women have significantly higher levels of sudden and constant anxiety than men. Therefore, they suggested developing behavior management strategies for women.

Signs and symptoms of inflammation, including pain, trismus and bleeding are common after impacted third molar surgeries. In terms of postoperative comfort, patients complain about pain the most (22). It is known that women and men have significantly different pain perception levels. Women feel more pain and react more negatively to pain compared to men (21). There are reports that men and women also differ in other inflammatory findings (23). Some studies suggested that psychological phenomena such as anxiety may lead to changes in inflammatory response (23-25). Additionally, sex hormones also alter vascular permeability and sensitivity of inflammatory mediators (26). Therefore, inflammatory responses, including pain and oedema may be expected to differ between the genders, regardless of the psychological causes (27). In parallel, in cases where the inflammatory response is severe, postoperative comfort and satisfaction levels can be expected to be low, regardless of the causative factor.

Benediktsdóttir *et al*. (28) used postoperative satisfaction and complication questionnaires similar to the ones used in this study to assess postoperative satisfaction levels and complications. Consequently, they found significantly higher rates of female patients complaining of severe trismus, pain and edema were significantly higher than male patients. They also found that female patients were more reluctant than male patients to have the same surgery again and to recommend it to others. These findings suggest that female patients experience complications more severely, possibly due to excessive levels of anxiety.

Eighty percent of women menstruate regularly. Hormonal changes during menstrual cycle lead to both psychological and physiological changes (18). The premenstrual period is characterized by a low pain threshold, as well as an increase in the incidence of edema, body temperature and anxiety levels. The incidence of asthma, migraine and epilepsy attacks also increase during this phase (7). In a clinical study, Gonda *et al*. (29) stated that obsessive-compulsive disorder and major depression were mostly triggered in the premenstrual period. Physical conditions such as fatigue, headache and backache often accompany the menstrual period along with a tense mood (7,19). The hormones secreted from the pituitary gland and ovaries lead to the menstruation through feedback mechanism (7). Progesterone and oestrogen hormones secreted from the ovaries determine the phase of the endometrium with clinical implications (7,18). In a review study published in 2015, Iacovides *et al*. (30) stated that only a few studies investigated the relationship between the menstrual cycle and pain perception. They argued that the physiological effects of the hormonal cycle occur mostly depending on progesterone and estrogen levels. This study’s findings demonstrated that the preoperative anxiety and fear levels were significantly elevated in the secretory phase, which also includes the premenstrual period when progesterone levels are high. On the other hand, the fact that female patients felt more comforTable in the proliferative phase may be attributed to the increasing estrogen levels in this phase. Postoperative satisfaction and complication results of female patients were also found to be parallel to their preoperative anxiety and fear levels. The physiological or psychological changes caused by postoperative inflammation might have influenced patients’ satisfaction levels.

In other studies investigating the effect of gender and menstruation-related hormones on pain levels, no significant results were obtained when the endometrial phase was not evaluated (30). Such findings may be attributed to not recognizing menstrual irregularity or inadequate sampling. On the other hand, the findings of studies featuring the clinical evaluation of the endometrial phase revealed significant results (20), in line with the findings of this study.

The strength of the study is that it is the first large-scale study to investigate the psychological and physiological consequences of hormonal differences in the context of oral surgery. However, the study also has some limitations. Firstly, the inclusion criterion of regular menstruation extended the study period to reach a sufficient number of patients. The second major limitation is the estimation of the menstrual cycle phase based on patient reports without gynaecological examination. Thirdly, the collection of data on patients' preoperative psychological state and postoperative symptoms was challenging as it required cooperation and close communication with the patients.

To conclude, female patients had higher preoperative anxiety and fear levels, lower postoperative satisfaction levels, and higher complication rates than male patients. Female patients in the proliferative menstrual cycle phase had significantly lower preoperative anxiety and fear levels, higher postoperative satisfaction levels, and lower complication rates than female patients in other menstrual cycle phases. On the other hand, female patients in the secretory phase had significantly higher preoperative anxiety and fear levels, lower postoperative satisfaction levels, and higher complication rates than female patients in other menstrual cycle phases. In conclusion, these findings suggest the importance of timing in reducing preoperative anxiety and fear levels and increasing postoperative satisfaction levels and complications rates in women. However, further studies are needed to determine the right timing and optimum conditions for a successful surgical dental procedure.

## Figures and Tables

**Table 1:** Postoperative complication questionnaire.

Questions
I had no difficulty eating after the operation?
I had no difficulty swallowing after the operation?
There was no change in my diet after the operation?
No change in my voice after the operation?
I had no difficulty in speaking after the operation?
When I talked to people after the operation, there was no problem in understanding me?
No bruising occurred in my head and neck area after the surgery?
There was no swelling in my head and neck area after the operation?
I was expecting the change that will occur after the surgery?
I was able to control the postoperative pain with analgesics?
The pain after the operation did not affect my daily life?
No weakness occurred after the operation?
I did not have nausea or vomiting after the operation?
My work/housework/daily activities were not affected due to the reasons caused by the surgery?
I did not have to take leave from work and leave my job unfinished due to tooth extraction?
I did not have any sleep problems after the operation?
I was satisfied with the tooth extraction treatment?
I would recommend this treatment to others?
If necessary, I will have the tooth extraction treatment done again?
I feel that my problem that caused me to seek treatment has been solved?

**Table 2:** Postoperative complication questionnaire.

Questions
I had pain after impacted wisdom tooth extraction.
Swelling occurred on my face after impacted wisdom tooth extraction.
I had bleeding after impacted wisdom tooth extraction.
I had difficulty swallowing after impacted wisdom tooth extraction.
Numbness occurred in my tongue after impacted wisdom tooth extraction.
Numbness occurred in the lower lip area after impacted wisdom tooth extraction.
After the extraction of impacted wisdom teeth, I think that my wound site has not healed and there is an opening.
After the extraction of impacted wisdom teeth, there was a restriction in my mouth opening.

**Table 3:** Distribution of the scores patients obtained from the preoperative anxiety and fear and postoperative satisfaction and complication assessments tools by the gender groups.

Variables	Female (n=79)	Male (n=49)	*p value*
	M[Min-Max]	M[Min-Max]	
STAİ total score	94[43-148]	63[52-151]	<0.001^a^
STAİ State	49[22-76]	33[20-80]	<0.001^a^
STAİ Trait Anxiety	48[21-73]	31[25-71]	<0.001^a^
DFS total score	58[20-94]	27[20-82]	<0.001^a^
MDAS total score	16[5-24]	9[5-25]	<0.001^a^
MDAS <19	50(51%)	48(49%)	<0.001^b^
MDAS ≥19	29(96.7%)	1(3.3%)	<0.001^b^
PSQ total score	116[58-123]	51[24-140]	<0.001^a^
PCQ total score	40[9-53]	13[9-28]	<0.001^a^

Abbreviations: STAI-S: State-Trait Anxiety Inventory Short Version, DFS: Dental Fear Survey, MDAS: Modified Dental Anxiety Scale, PSQ: postoperative satisfaction questionnaire, PCQ: postoperative complication questionnaire. Data are given as median with minimum and maximum M: Median, a: Mann Whitney U test, b: Pearson's Chi-Square test.

**Table 4:** Distribution of the scores female patients obtained from the preoperative anxiety and fear and postoperative satisfaction and complication assessments tools by the menstrual cycle phases.

Variables	Menstrual ^1^ (n=30)	Proliferative ^2^ (n=26)	Secretory ^3^ (n=23)	*p value* ^c^	Paired Comparison
	M[Min-Max]	M[Min-Max]	M[Min-Max]		
STAİ total score	100.5[43-131]	87[59-120]	147[65-148]	<0.001	*p* ^1-2^: 0.005
					*P* ^2-3^: <0.001
					*p* ^1-3^: <0.001
STAİ State	54.5[22-67]	43[23-59]	74[33-76]	<0.001	*p* ^1-2^: 0.005
					*P* ^2-3^: <0.001
					*p* ^1-3^: <0.001
STAİ Trait Anxiety	49[21-68]	44[30-61]	72[32-73]	<0.001	*p* ^1-2^: 0.034
					*P* ^2-3^: <0.001
					*p* ^1-3^: <0.001
DFS total score	67.5[20-94]	50[20-78]	88[22-94]	<0.001	*p* ^1-2^: 0.100
					*P* ^2-3^: <0.001
					*p* ^1-3^: <0.001
MDAS total score	17[5-24]	12[5-19]	20[5-24]	<0.001	*p* ^1-2^:0.020
					*P* ^2-3^: <0.001
					*p* ^1-3^: 0.011
PSQ total score	51[40-140]	79[32-102]	35[24-80]	<0.001	*p* ^1-2^: 0.001
					*P* ^2-3^: <0.001
					*p* ^1-3^: <0.001
PCQ total score	42[15-45]	22[19-40]	49[9-53]	<0.001	*p* ^1-2^: <0.001
					*P* ^2-3^: <0.001
					*p* ^1-3^: <0.001

Abbreviations: STAI-S: State-Trait Anxiety Inventory Short Version, DFS: Dental Fear Survey, MDAS: Modified Dental Anxiety Scale, PSQ: postoperative satisfaction questionnaire, PCQ: postoperative complication questionnaire. Data are given as median with minimum and maximum M: Median, c: Kruskal Wallis H Test.
